# Nuclear actin modulates cell motility via transcriptional regulation of adhesive and cytoskeletal genes

**DOI:** 10.1038/srep33893

**Published:** 2016-09-21

**Authors:** Amir S. Sharili, Fiona N. Kenny, Maria K. Vartiainen, John T. Connelly

**Affiliations:** 1 Centre for Cell Biology and Cutaneous Research, Barts and the London School of Medicine and Dentistry, Queen Mary University of London, London E1 2AT, U.K.; 2 Institute of Biotechnology, University of Helsinki, 00790 Helsinki, Finland; 3 Institute of Bioengineering, Queen Mary University of London, London E1 2AT, U.K.

## Abstract

The actin cytoskeleton is a classic biomechanical mediator of cell migration. While it is known that actin also shuttles in and out of the nucleus, its functions within this compartment remain poorly understood. In this study, we investigated how nuclear actin regulates keratinocyte gene expression and cell behavior. Gene expression profiling of normal HaCaT keratinocytes compared to HaCaTs over-expressing wild-type β-actin or β-actin tagged with a nuclear localization sequence (NLS-actin), identified multiple adhesive and cytoskeletal genes, such as *MYL9*, *ITGB1*, and *VCL,* which were significantly down-regulated in keratinocytes with high levels of nuclear actin. In addition, genes associated with transcriptional regulation and apoptosis were up-regulated in cells over expressing NLS-actin. Functionally, accumulation of actin in the nucleus altered cytoskeletal and focal adhesion organization and inhibited cell motility. Exclusion of endogenous actin from the nucleus by knocking down Importin 9 reversed this phenotype and enhanced cell migration. Based on these findings, we conclude that the level of actin in the nucleus is a transcriptional regulator for tuning keratinocyte migration.

Actin is an essential component of the cytoskeleton, which supports overall cell structure, adhesion to the extracellular matrix, and the generation of contractile forces through interaction with myosin motor proteins^1^,^2^. While its mechanical and structural functions have been studied in depth, relatively little is known about the role of actin in the nucleus.

Actin has been known in to exist in the nucleus for many years^3^,^4^, but only recently have the molecular mechanisms that control its localization and function within this compartment been investigated. It is now clear that actin dynamically shuttles in and out of the nucleus^5^ through the activity of Importin 9^6^ and Exportin 6^7^, respectively, and as it lacks a nuclear localization sequence (NLS), actin requires specific co-factors, such as cofilin, for transport across the nuclear membrane^6^. Nuclear actin comprises predominantly monomeric G-actin, but can form F-actin filaments under specific conditions. For example, high levels of F-actin are present in *Xenopus laevis* oocytes and are required for reprogramming^8^,^9^. In addition, actin filaments rapidly form in the nucleus following serum stimulation via the formin, mDia^10^, and actin oligomers can interact with the nuclear receptor co-repressor complex^11^.

Nuclear actin mediates several key transcriptional processes. It physically interacts with all three RNA polymerases and helps stabilize the pre-transcriptional complex^12^,^13^. It interacts with chromatin remodeling complexes, such INO80 and BAF^14^,^15^, and nuclear G-actin negatively regulates the transcriptional activity of serum response factor (SRF) by binding and inhibiting the co-factor megakaryoblastic leukemia 1 (MKL1)^16^,^17^. While some of the basic functions of actin in the nucleus have been described, only a few studies have linked these effects to changes in cell behavior. In mammary epithelial cells, depletion of nuclear actin via adhesion to laminin induces quiescence^18^, while accumulation of nuclear actin in mesenchymal stem cells promotes osteogenic gene expression^19^. Thus, there is clear evidence that nuclear actin regulates important cellular processes, but its complete set of functions has yet to be described.

The aim of the present study was to determine the role of nuclear actin in the regulation of human keratinocyte behavior. Through mRNA expression profiling, we demonstrate that nuclear actin negatively regulates a core set of adhesive and cytoskeletal genes and inhibits cell motility. Together, these results provide new insights into the broad range of functions of nuclear actin and identify a novel transcriptional mechanism by which actin regulates cell migration and motility.

## Results

### Nuclear actin negatively regulates expression of adhesive and cytoskeletal genes

**Figure 1:**
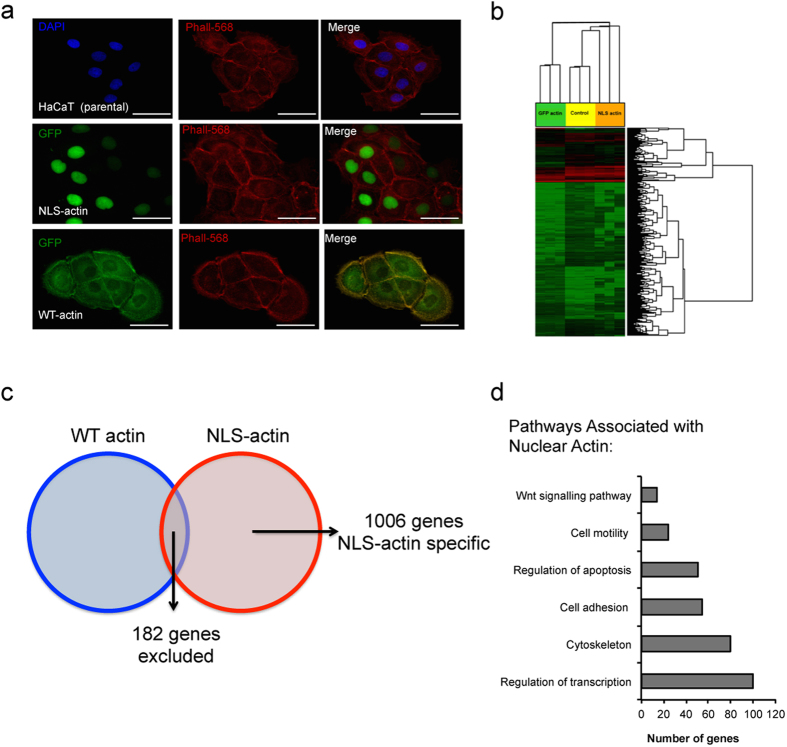
(**a**) Representative images of parental HaCaT keratinocytes and cell lines stably expressing NLS-actin or WT-actin tagged with GFP. F-actin was stained with phalloidin (red) and nuclei were labeled with DAPI (blue). Scale bars are 50 μm (**b**) Hierarchical clustering of genes associated with HaCaT (parental), WT-actin, or NLS-actin cells. (**c**) Venn diagram illustrating nuclear actin-specific genes selected for pathway analysis. (**d**) Example of Gene Ontology terms and numbers of genes that are uniquely differentially expressed in NLS-actin cells compared to the parental line. Analysis was performed using the DAVID on-line resource.

To gain insight into how nuclear actin regulates cell function, we generated HaCaT keratinocyte lines stably expressing wild type β-actin-GFP (WT-actin) or β-actin-GFP tagged with a nuclear localization sequence (NLS-actin), which forces actin to accumulate within the nucleus (Fig. 1a). There were no detectable effects of NLS-actin overexpression on HaCaT growth, differentiation, or general morphology (see Supplementary Figure S1). We performed gene expression profiling of the parental HaCaT cells, WT-actin cells, and NLS-actin cells using the Affymetrix 2.0 microarray platform (Fig. 1b). Analysis of genes with >2.0-fold (P < 0.05) changes in expression revealed 1188 genes differentially expressed between NLS-actin cells and the parental HaCaT line (GEO accession number GSE78028). Within this set, 182 genes were similarly affected by WT-actin and therefore excluded from further analysis as non-specific effects of actin or GFP overexpression (Fig. 1c). Pathway analysis of the remaining 1006 nuclear actin-specific genes was performed using the Database for Annotation, Visualization, and Integrated Discovery (DAVID) platform, and identified ‘Regulation of transcription’, ‘Cytoskeleton’, ‘Cell adhesion’, and ‘Motility’ as Gene Ontology terms enriched in this set of genes (Fig. 1d).

**Figure 2:**
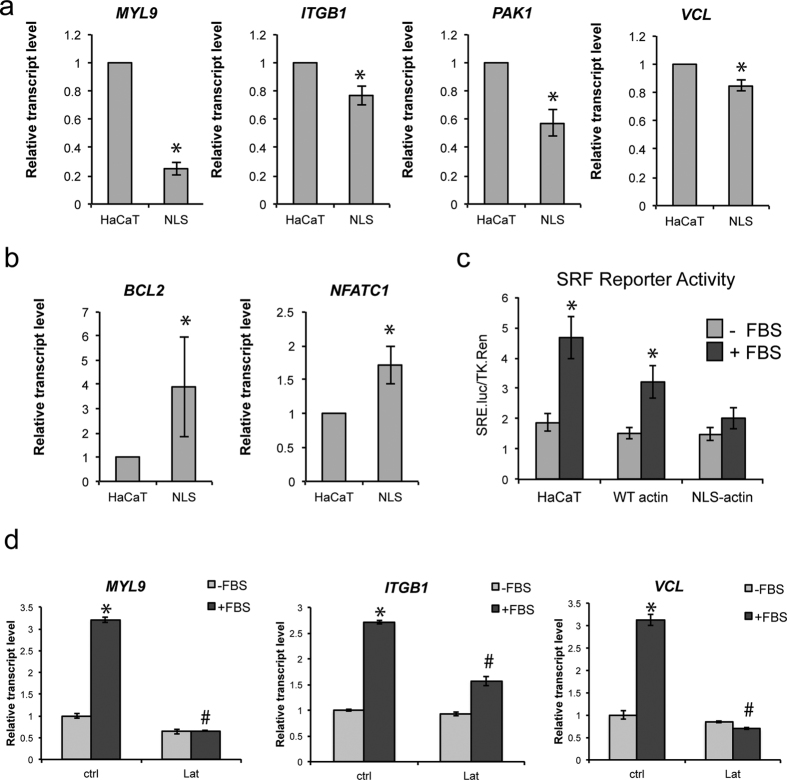
Nuclear actin regulates specific patterns of gene expression. (**a**) QRT-PCR validation of selected genes significantly down-regulated or (**b**) up-regulated in the NLS-actin cells compared to the HaCaT parental line. Expression levels are relative to *GAPDH* and normalized to HaCaT levels. Data represent the mean ± SEM of 3 experiments. *P < 0.0001 (*MYL9*), P = 0.030 (*ITB1*), P = 0.012 (*PAK1*), P = 0.015 (*VCL*), P = 0.03 (*NFATC1*), P = 0.039 (*BCL2*). (**c**) SRF transcriptional activity in HaCaT, WT-actin, and NLS-actin cells following stimulation with 10% FBS for 7 h. Data are expressed as SRF luciferase activity relative Renilla control and represent the mean ± SEM of 3 experiments. *P = 0.0003 for −FBS vs + FBS (HaCaT), P = 0.014 for −FBS vs + FBS (WT-actin). (**d**) Analysis of SRF targets *MYL9*, *ITGB1*, and *VCL* by qPCR in HaCaT cells following 4 h treatment with 10% FBS or 1 μM Latrunculin A (Lat). Expression levels are relative to *GAPDH* and normalized to untreated control (ctrl) levels. Data represent the mean ± SEM of 3 replicates. *P < 0.001 compared to ctrl −FBS, ^#^P < 0.001 compared to ctrl + FBS.

Within these categories, several known regulators of cell adhesion and motility, such as *ITGB1*, *MYL9*, *PAK1*, and *VCL*, were all significantly down-regulated in the NLS-actin expressing cells, and these results were confirmed by qRT-PCR (Fig. 2a). We also confirmed the up-regulation of specific genes, such as *NFATC1* and *BCL2*, in the NLS-actin cells by qRT-PCR (Fig. 2b). We further verified that these changes in gene expression were specific to actin not a consequence of GFP accumulation in the nucleus by comparing gene expression in HaCaT cells stably expressing the nuclear GFP chromobody^20^ compared to NLS-actin cells (see Supplementary Figure S2).

Given that several of the down-regulated genes are known SRF targets^21^,^22^,^23^, we also measured SRF transcriptional activity using a luciferase reporter construct. SRF activity was significantly increased with FBS stimulation in the parental and WT-actin cells but not in the NLS-actin cells (Fig. 2c). Consistent with being SRF target genes, *MYL9*, *ITGB1*, and *VCL* were all up-regulated in HaCaT cells following serum stimulation, and this response could be blocked by disrupting actin polymerization with Latrunculin A (Fig. 2d). We also tested how changes in gene expression depended on the polymerization status of nuclear actin by comparing the effects of NLS-actin overexpression to overexpression of the non-polymerizable actin mutant, R62D^24^, also tagged with NLS. Although actin filaments were occasionally (<0.1%) observed in the NLS-actin cells, both cell lines displayed a similar down-regulation of *ITGB1* and *VCL* and up-regulation of *NFATC1*, indicating that the effects of NLS-actin on HaCaT gene expression did not depend on polymerization (see Supplementary Figure S2). Together, these results suggest that, nuclear actin inhibits expression of adhesive and cytoskeletal genes via impaired transcriptional activity of SRF.

### Nuclear actin alters cytoskeletal organization and inhibits cell migration

**Figure 3:**
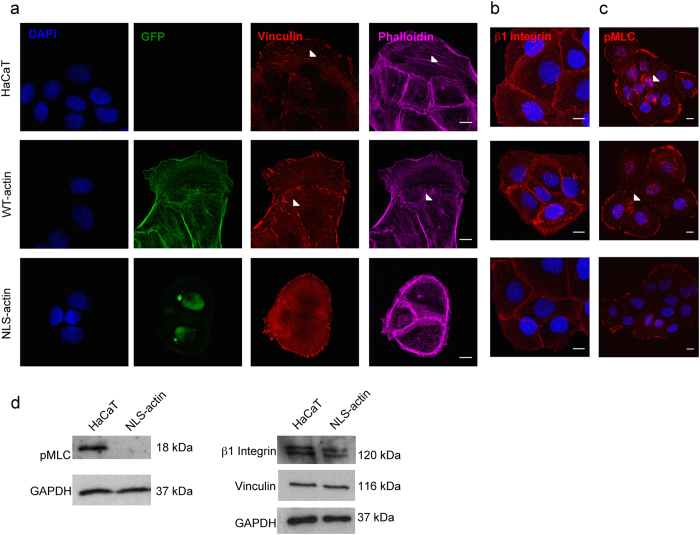
Accumulation of actin in the nucleus disrupts cytoskeletal organization. (**a**) Representative confocal fluorescence images of GFP, vinculin, and F-actin in HaCaT, WT-actin, and NLS-actin lines. (**b**) Representative confocal fluorescence images of β1 integrin and (**c**) pMLC. Reduced focal adhesions, stress fibers, and pMLC could be observed in the NLS-actin cells, particularly on the interior of cell clusters (denoted by arrowheads in HaCaT and WT-actin cells). Scale bars are 10 μm. (**d**) Western blot analysis of total β1 integrin, phospho-myosin light chain (pMLC), and vinculin in HaCaT and NLS-actin lines. GAPDH was used as a loading control.

To determine whether the changes in mRNA expression in NLS-actin cells correlated with protein level changes, we examined the expression and localization of the β1 integrin, vinculin, and phospho-myosin light chain (pMLC, activated myosin) by immunofluorescence imaging and Western blot analysis. Compared to the parental and WT-actin lines, NLS-actin expressing keratinocytes displayed reduced stress fibers and vinculin-containing focal adhesions, particularly within the interior of multi-cell clusters (Fig. 3a). In addition, NLS-actin cells expressed lower overall levels of the β1 integrin (Fig. 3b), and there was reduced pMLC (Fig. 3c). Western blot analysis confirmed the reduced levels of β1 integrin and pMLC in NLS-actin cells compared to the parental HaCaTs (Fig. 3d), but the total amount of vinculin was similar in the HaCaT and NLS-actin cells (Fig. 3d). Thus, down-regulation of *MYL9* and *ITGB1* at the gene level corresponds with reduced contractility and total β1 integrin protein expression. As decreased *VCL* expression did not alter total vinculin protein levels in NLS-actin cells, the observed changes in focal adhesion assembly were most likely due to reduced contractility and cytoskeletal tension. Overall, we conclude that accumulation of actin in the nucleus disrupts focal adhesion assembly, cytoskeletal organization, and acto-myosin contractility.

**Figure 4:**
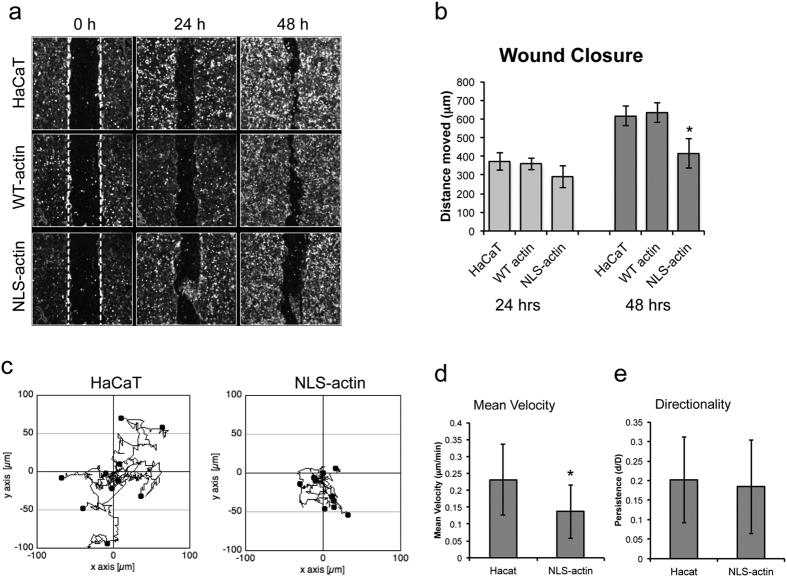
Nuclear actin inhibits cell motility. (**a**) Representative images (inverted phase contrast) of scratch wound assays at 0, 24, and 48 hours after wounding. (**b**) Quantification of wound closure by calculating the average distance moved per field of view (total scratch area divided by length of field of view). Data represent mean ± SEM of 3 experiments. *P = 0.0031 compared to WT-actin. (**c**) Tracks of individual cells in a single field of view over a 24 hour period for the HaCaT and NLS-actin lines. (**d**) Quantification of mean cell velocity. (**e**) Quantification of persistence (net displacement/total distance traveled) calculated using the Manual Tracking plug-in on ImageJ. Data represent mean ± SD of 30 cells. *P < 0.0001 compared to HaCaT.

Because adhesion and contractility are essential for cell motility, we next investigated whether altered cytoskeletal organization had a functional impact on keratinocyte migration. In scratch assays, wound closure was significantly lower after 48 hours for NLS-actin keratinocytes compared to the parental and WT-actin lines (Fig. 4a,b). This impaired migration could be qualitatively observed in time-lapse movies of NLS-actin cells (see Supplementary Movies S1 and S2), particularly in cells behind the leading edge where reduced stress fibers and pMLC were most prominent (Fig. 3a,c). We further quantified the effects of nuclear actin on cell motility by tracking single cells randomly migrating on collagen-coated coverslips (see Supplementary Movies S3 and S4). Compared to the parental control, NLS-actin cells displayed reduced migration speed but not directionality (Fig. 4c–e). Transient transfection of HeLa cells with NLS-actin expression vectors also significantly inhibited scratch wound closure, indicating that these effects were not limited to the HaCaT cell type or the mutant lines generated for these studies (see Supplementary Figure S3). We also confirmed that this response was specific to actin in the nucleus, rather than GFP in the nucleus, and similar to the effects on gene expression, a similar inhibition was observed in the NLS-R62D cells (see Supplementary Figure S3). Together, these results demonstrate that nuclear actin negatively regulates cell motility.

### Exclusion of actin from the nucleus enhances cell migration

**Figure 5:**
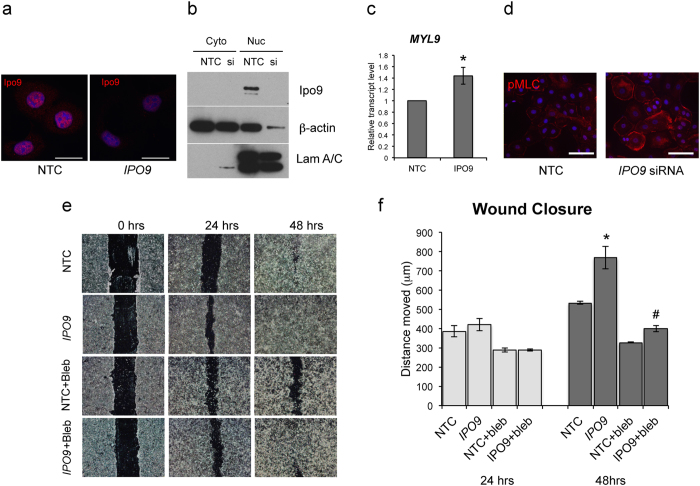
Knockdown of *IPO9* excludes actin from the nucleus and enhances cell migration. (**a**) Immunofluorescence images of Importin 9 (red) in HaCaT cells treated with non-targeting control (NTC) or *IPO9* siRNA for 48 hours. Scale bars are 25 μm. (**b**) Western blot analysis of Importin 9, β-actin, and Lamin A/C levels in nuclear and cytoplasmic fractions of cells treated with NTC or *IPO9* siRNA. (**c**) QRT-PCR measurement of *MYL9* expression relative to *GAPDH.* Data are normalized to NTC control and represent the mean ± SEM of 3 experiments. *P = 0.023 (**d**) Immunofluorescence detection of phosphorylated myosin light chain (pMLC) in NTC or siRNA treated samples. Scale bars are 50 μm. (**e**) Representative images (inverted phase contrast) of scratch wound closure at 0, 24, and 48 hours after wounding. Cells were treated with NTC or *IPO9* siRNA 48 hours prior to wounding, and cultured with either 0.1% DMSO or 50 μM blebbistatin after wounding. (**f**) Quantification of wound closure by calculating the average distance moved (total scratch area divided by length of the field of view). Data represent the mean ± SEM of 3 experiments. *P = 0.024 compared to NTC at 48 h, ^#^P = 0.023 compared to *IPO9* siRNA at 48 h.

Finally, we examined whether excluding actin from the nucleus could reverse the effects on keratinocyte behavior. We used siRNA to knock down the gene for Importin 9 (*IPO9*) and block actin from entering the nucleus. Knockdown of *IPO9* in HaCaT keratinocytes resulted in reduced levels of actin in the nucleus (Fig. 5a,b), increased expression of *MYL9* mRNA and increased pMLC staining (Fig. 5c,d). Furthermore, *IPO9* knockdown significantly increased scratch wound closure under normal culture conditions (Fig. 5e,f, see Supplementary Figure S4). As *MYL9* codes for a non-muscle myosin regulatory light chain required for cell contractility, we used the myosin inhibitor, blebbistatin, to functionally block this response (Fig. 5e,f). These experiments suggest that in addition to overexpression of NLS-actin, endogenous levels of nuclear actin are capable of affecting keratinocyte migration. While *IPO9* knock down may affect the transport of other molecules into the nucleus, the results are consistent with overexpression studies and suggest that the levels of actin in the nucleus both positively and negatively regulate cell migration.

## Discussion

The aim of this study was to determine the specific functions of nuclear actin in keratinocytes. Together, our data indicate that changes in nuclear actin modulate cell motility via coordinated transcriptional regulation of adhesive and cytoskeletal genes. Accumulation of actin in the nucleus led to decreased expression of *VCL*, *ITGB1*, and *MYL9*, altered cytoskeletal organization, and reduced cell migration speed and scratch wound closure. Conversely, exclusion of actin from the nucleus was associated with enhanced cell migration.

Actin is typically considered a mechanical regulator of cell migration. F-actin polymerization is essential for the protrusion of lamellipodia^25^, while tensile forces generated by F-actin stress fibers and myosin motor proteins facilitate cell contractility^1^. In addition to these mechanical functions, our results now provide evidence that actin can also affect cell migration via changes in gene expression. This response could potentially act as a positive feedback mechanism to reinforce or restrain cell migration within different adhesive microenvironments. For example, accumulation of actin in the nucleus following de-polymerization of the F-actin cytoskeleton^5^, could lead to down-regulation of adhesive and cytoskeletal genes and reduced cell motility.

Previous studies have shown that one of actin’s major transcriptional functions is the regulation of SRF activity^10^,^16^. In this instance, nuclear G-actin acts a transcriptional repressor by binding and inhibiting the function of SRF’s cofactor, MKL1. Consistent with previous studies, we find that accumulation of actin in the nuclei of keratinocytes also inhibits SRF activity. As several of the key adhesive genes (*MYL9*, *VCL*, *ITGB1*) are known to be direct SRF targets^21^,^22^,^23^, nuclear actin most likely inhibits their expression by interfering with SRF activity. At this time, however, we cannot rule out the potential effects of non-SRF target genes on cell motility. Our findings further suggest that nuclear actin in keratinocytes is predominantly monomeric; however, an important question for future work will be to directly measure the levels of polymerized versus non-polymerized actin in the nucleus and to determine their distinct activities.

Beyond the negative regulation of adhesion and cell migration, the effects of nuclear actin on other genes and transcriptional processes will be of significant interest in future studies. Our microarray analysis identified several genes, such as *NFATC1* and *BCL2*, which were significantly up-regulated by nuclear actin. In addition, functional categories including ‘transcription’, ‘apoptosis, and ‘Wnt signaling’ were associated with increased nuclear actin. These genes and pathways may reflect additional functions of actin in the nucleus. For example, *NFATC1* regulates epidermal stem cell quiescence^26^, and recent studies indicate that nuclear actin polymerization facilitates DNA damage responses^27^. As F-actin polymerization and cytoskeletal organization are sensitive to extracellular biophysical cues, the effects of upstream mechanical and adhesive signals on nuclear actin localization will also be an important area of investigation in future studies.

## Methods

### Cell culture and generation of stable cell lines

HeLa and HaCaT^28^ cell lines were maintained in Dulbecco’s modified Eagle’s medium (DMEM) supplemented with 10% (v/v) fetal bovine serum, 100 units/ml penicillin, and 100 mg/ml streptomycin. Stable HaCaT cell lines were generated by transfection with linearized plasmids for EGFP-actin (Clontech, Mountain View, CA), nuclear actin chromobody (Chromotek, Martinsried, Germany), NLS-EGFP-actin, or NLS-EGFP-R62D (kindly provided by Dr Maria Vartiainen, University of Helsinki)^6^. Cells were transfected using Lipofectamine 2000 according to the manufacturers instructions and selected with 0.5 mg/ml G418 until a stable GFP positive population was established. All cell culture reagents were from Thermo Fisher Scientific unless otherwise stated.

### Scratch assays and cell migration

Confluent cell monolayers in a 12 well plate were scratched with a 200 μl pipette tip to obtain two perpendicular wounds, forming a cross shape. Wounds were imaged at 0, 24 and 48 hours using a Nikon Eclipse TE2000-5 microscope (Nikon, Kingston Upon Thames, UK). Average distances between wound edges were calculated by measuring the uncovered wound area and dividing by the length of the field of view. Distance migrated was calculated by subtracting the average distance between wound edges from the distance at time 0. For each experiment a total of 12 wounds were measured per group. Scratch closure and random cell migration was also monitored using time-lapse microscopy every 30 minutes for 24 hours with a Zeiss Axiovert 200 M microscope (Zeiss, Oberkochen, Germany). For random cell migration analysis, single cells were tracked across 3 fields of view per condition, and mean migration speed and persistence were calculated for 10 cells per field using ImageJ and the Manual Tracking plug-in.

### Gene expression profiling

Total RNA was extracted using Agilent Absolutely RNA miniprep kit (Agilent Technologies, Stockport, UK), followed by cRNA probe generation (Ovation V2: Nugen Technolgies Inc, San Carlos, CA) and hybridization to the human U133 Plus 2.0 arrays (Affymetrix, Santa Clara, CA) containing over 54000 probe sets for 47000 human transcripts and variants. The array slides were scanned with a GeneChip Scanner 3000 and the CEL files were processed using the MAS5 algorithm. For each group (HaCaT, GFP-actin and NLS-GFP-actin) three experimental replicates were used. Differential gene expression was determined using a p-value cut off of 0.05 and fold change of >2. Gene Ontology terms were identified by analyzing the differentially expressed genes using the Functional Annotation tool within the Database for Annotation, Visialization, and Integrated Discovery. Microarray data are freely available through the NCBI Gene Expression Omnibus, accession number GSE78028.

### Quantitative RT-PCR

Total RNA was isolated from cells using the RNeasy Plus Mini Kit (Qiagen, Manchester, UK). cDNA was synthesized from 1 μg of total RNA using Superscript III Reverse transcriptase (Thermo Fisher Scientific). Quantitative real-time RT-PCR (qRT-PCR) was carried out using Rotor-Gene SYBR green PCR kit (Qiagen) and Rotor-Gene Q PCR cycler (Qiagen). Primers used were against *ITGB1*, *MYL9*, *VCL*, *PAK1*, *NFATC1*, *BLC2* and the housekeeping gene *GAPDH* (see Supplementary Table S1). The mRNA expression level of each gene relative to GAPDH was calculated using the ΔΔCt method.

### Immunofluorescence staining and imaging

Cells were fixed in 4% PFA for 10 minutes and permeabilized with 0.2% Triton-X100. Cells were blocked for 45 min in 2% bovine serum albumin and incubated with primary antibody overnight at 4 °C. Primary antibodies used were mouse anti-vinculin (1:200, hVIN-1, Sigma Aldrich), mouse anti-β1 integrin (1:200, P5D2, Cancer Research UK, London, UK), rabbit anti-phospho-myosin light chain (1:100, Cell Signaling, Danvers, MA), and rabbit anti-Importin 9 (1:100, Abnova, Taipei, Taiwan). Coverslips were washed and incubated for 60 minutes with goat anti-mouse or goat anti-rabbit secondary antibody Alexa Fluor^®^ 568 (1:1000, Thermo Fisher Scientific) and DAPI. Alexa Fluor 568- or 633-phalloidin (Thermo Fisher Scientific) staining was used to visualize F-actin. Imaging was performed with a Zeiss 710 confocal microscope.

### Luciferase reporter assay

Cells were transfected with the SRF luciferase reporter (p3DA.luc) using Lipofectamine 2000 and 1 μg of DNA per 10^5^ cells as previously described^29^,^30^. Thymidine kinase driven Renilla was used as an internal control and transfected 1:1 with p3DA.luc. Following DNA transfection, cells were rinsed and cultured for 24 hours before treating with FBS. Luciferase assays were carried out in 24-well plates (n = 4 wells); cells were treated for 7 hours with FBS, then harvested and analyzed using the dual luciferase assay (Promega, Southampton, UK).

### Western blot analysis

Cells were washed in PBS, scraped off the dish, and either lysed in RIPA for whole cell protein or incubated in a hypotonic buffer (10 mM HEPES, 1.5 mM MgCl_2_, 10 mM KCl plus protease inhibitors) for 10 min on ice for nuclear fractionation. Cells for fractionation were then lysed by with a dounce, and the nuclear and cytoplasmic fractions were separated by centrifugation. The nuclear pellet was resuspended in RIPA plus protease inhibitors (Roche, Burgess Hill, UK). Protein concentration was determined by the BCA assay (Thermo Fisher Scientific). Lysates were combined with loading buffer (Thermo Fisher Scientific) and 1% β2-Mercaptoethanol (Sigma Aldrich), and 10 μg of total protein was resolved on a 10% polyacrylamide gel (Bio-Rad, Hemel Hempsted, UK) and transferred onto nitrocellulose membranes (GE Lifesciences). Membranes were blocked for 1 h in 5% non-fat dry milk, before being incubated with primary antibody for mouse anti-vinculin (1:10,000, hVIN-1, Sigma Aldrich), mouse anti-β1 integrin (1:1000, P5D2, Cancer Research UK, London, UK), rabbit anti-phospho-myosin light chain (1:1000, Cell Signaling, Danvers, MA), rabbit anti-GAPDH (1:5000 Abcam, Cambridge UK), rabbit anti-Importin 9 (1:1000; Abnova, Taipei, Taiwan), rabbit anti-β-actin (Abcam, Cambridge, UK) and mouse Lamin A/C (1:1000, Santa Cruz Biotechnology, Santa Cruz, CA) overnight at 4 °C. Secondary detection was performed with a HRP conjugated anti-rabbit or anti-mouse antibodies (1:5000, Thermo Fisher Scientific). Proteins were visualized using the enhanced chemiluminescence detection system (Millipore, Watford, UK).

### siRNA knockdown

Non-targeting control siRNA and human *IPO9* Silencer Select siRNAs (siRNA ID s31299 (si1) and s31300 (si2)) were purchased from Thermo Fisher Scientific (Ambion). HaCaT cells were transfected with 20 pmol RNA per 10^5^ cells using Lipofectamine 2000. Cells were seeded onto 12-well plates or 13 mm coverslips 2 days after transfection for scratch assays or immunofluorescence imaging, respectively.

### Statistics

Data are presented as mean ± SD for single cell measurements (random cell migration) or mean ± SEM for population level measurements (qPCR and wound closure). Data were tested for normality using the Anderson-Darling test. Statistical analysis was performed using one-way ANOVA and Tukey’s test for post-hoc analysis, and significance was determined by P < 0.05.

## Additional Information

**How to cite this article**: Sharili, A. S. *et al.* Nuclear actin modulates cell motility via transcriptional regulation of adhesive and cytoskeletal genes. *Sci. Rep.* **6**, 33893; doi: 10.1038/srep33893 (2016).

## Supplementary Material

Supplementary Information

Supplementary Movie 1

Supplementary Movie 2

Supplementary Movie 3

Supplementary Movie 4
