# Supplementary material for: Nuclear actin modulates cell motility via transcriptional regulation of adhesive and cytoskeletal genes
Source: Sci Rep. 2016 Sep 21;6:33893. doi: 10.1038/srep33893 (PMC5030641; doi:10.1038/srep33893)
Supplement: Supplementary Information [file srep33893-s1.pdf]

Nuclear actin modulates cell motility via transcriptional regulation of adhesive and cytoskeletal genes

Amir S. Sharili[1], Fiona N. Kenny[1], Maria K. Vartiainen[2], John T. Connelly*[1,3]

1. Centre for Cell Biology and Cutaneous Research, Barts and the London School of Medicine and Dentistry, Queen Mary University of London, London E1 2AT, U.K.
2. Institute of Biotechnology, University of Helsinki, 00790 Helsinki, Finland.
3. Institute of Bioengineering, Queen Mary University of London, London E1 2AT, U.K.

Supplementary data:

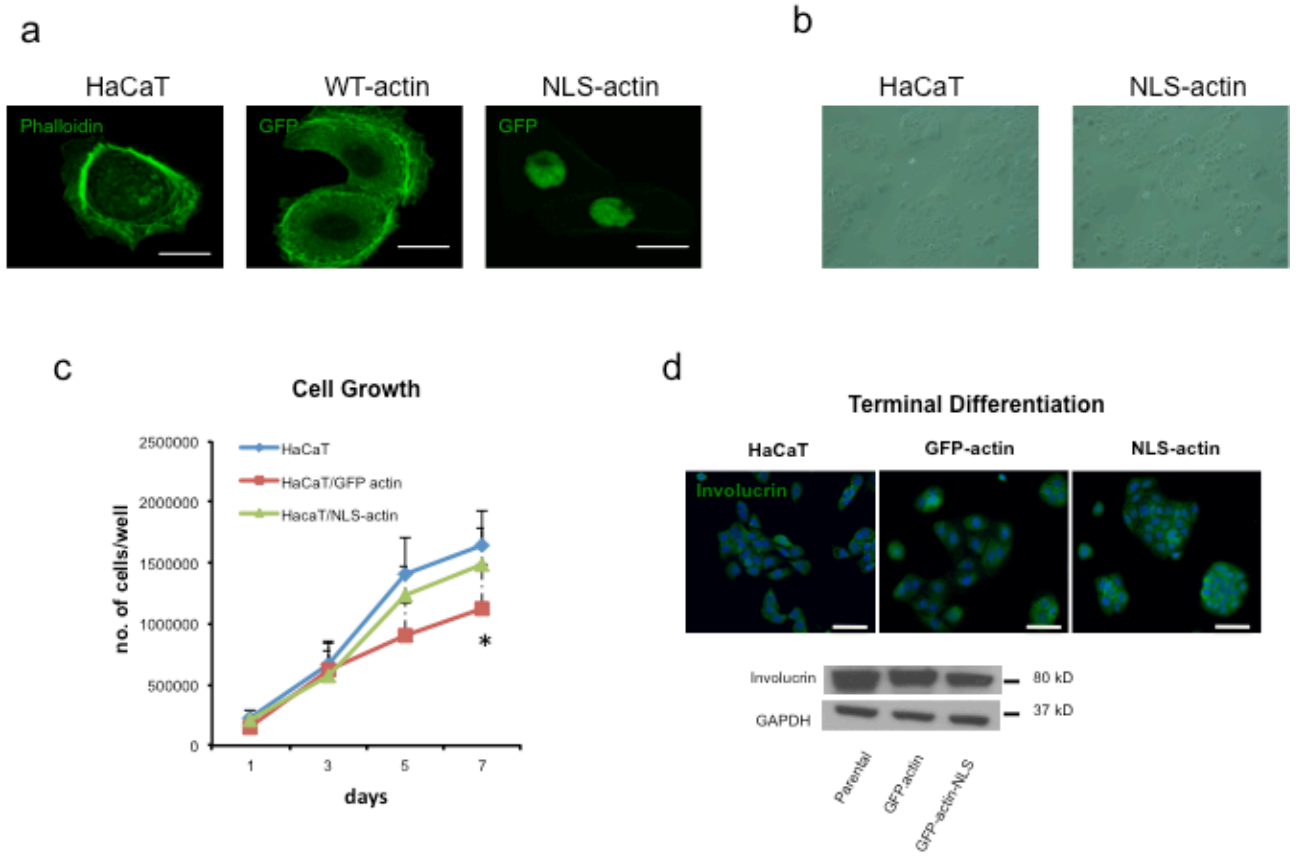

Figure S1: Phenotypic characterization of HaCaT cell lines. (a) Fluorescence images of F-actin in parental HaCaT cells and GFP in HaCaT cells stably expressing GFP-WT-actin and GFP-NLS-actin. Scale bars equal 25 μm. (b) Phase contrast images of HaCaT and NLS-actin lines showing similar epithelial morphologies. (c) Quantification of cell growth over 7 days for HaCaT, WT-actin, and NLS-actin cell lines. At each time point cells were trypsinised and counted from triplicate wells of 6-well plates. $^*P<0.05$ HaCaT vs WT-actin (N=3). (d) Immunofluorescence images and Western blot analysis of involucrin expression following 48h stimulation with 1.8 mM Ca^{++}. Scale bars equal 100 μm.

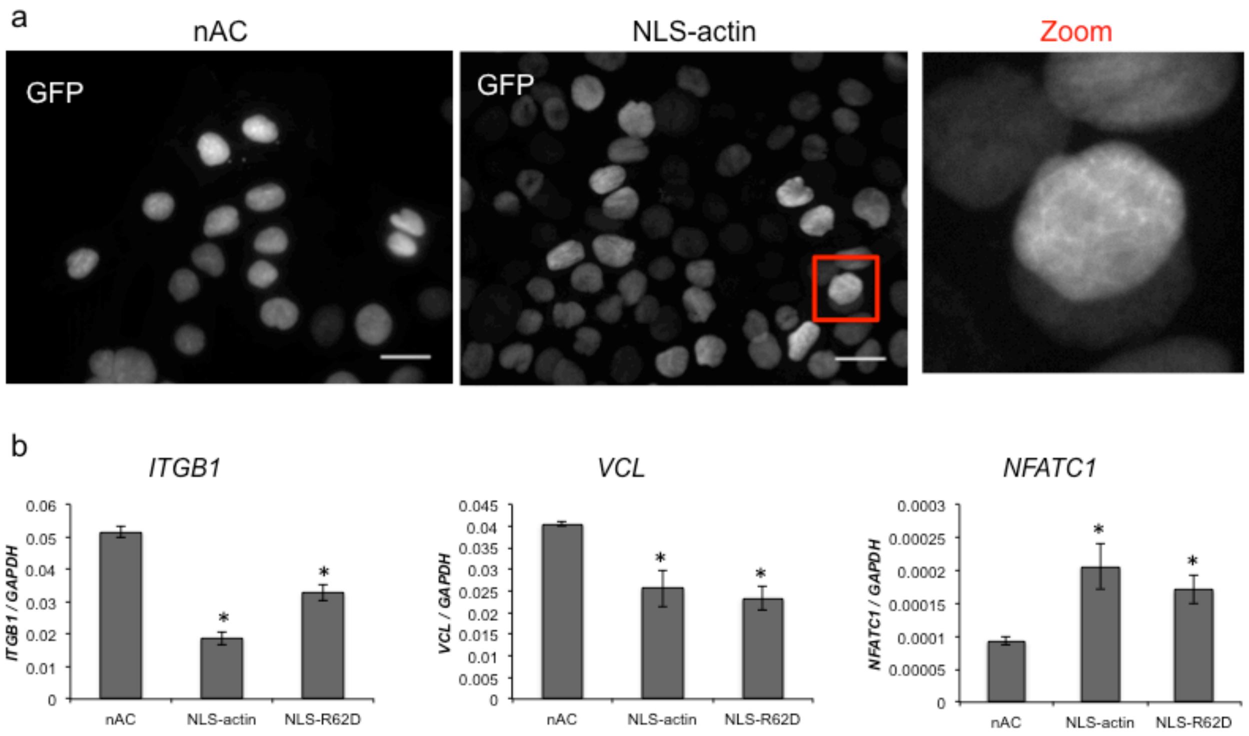

Figure S2: Specificity of nuclear actin effects on gene expression. (a) Representative fluorescence images of HaCaT cells stably expressing the nuclear anti-actin GFP chromobody (nAC) or NLS-actin. Zoom shows a rare (<0.01%) cell with nuclear F-actin filaments. Scale bars equal 25 µm. (b) Quantification of gene expression for *ITGB1*, *VCL*, and *NFATC1* by qPCR in HaCaT lines expressing nAC, NLS-actin, or NLS-R62D (non-polymerisable actin mutant). Expression is normalized to *GAPDH* and data represent mean ± SEM, *P<0.05 compared to nAC controls (N=3).

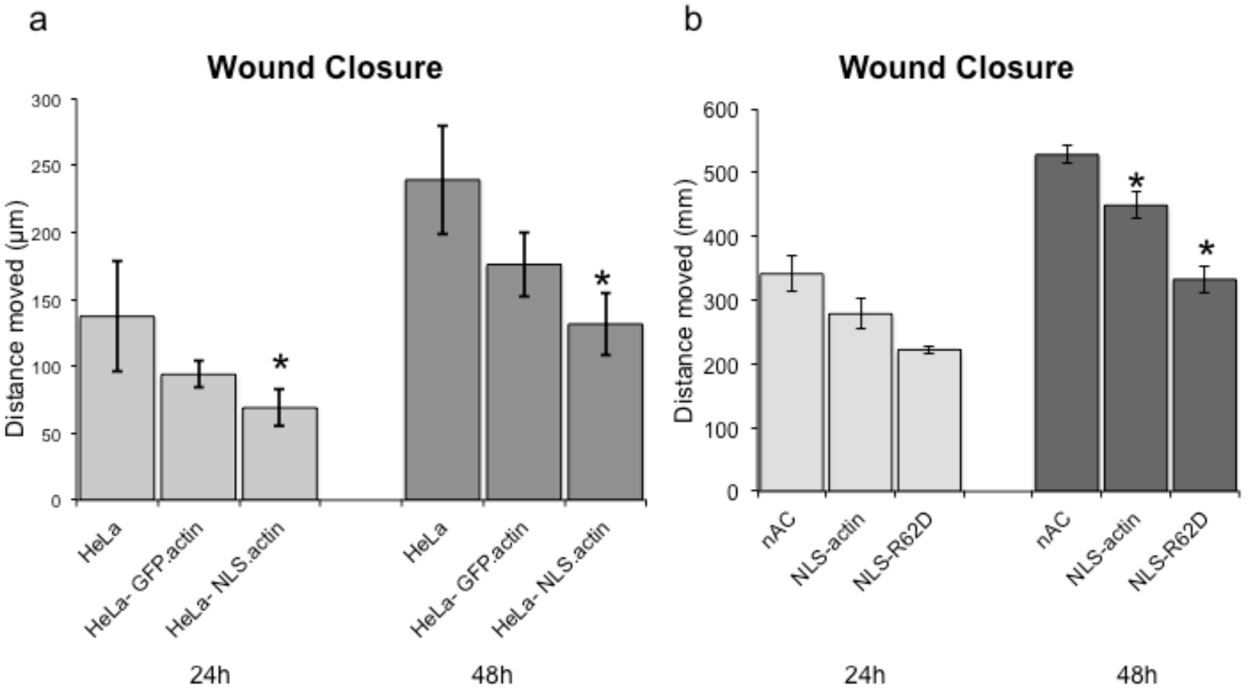

Figure S3: Specificity of nuclear actin effects on migration. (a) Quantification of scratch wound closure of non-transfected HeLa cells and cells transfected with WT GFP-actin or NLS-actin. (b) Quantification of scratch wound closure in HaCaT cells expressing the nuclear actin chromobody (nAC), NLS-actin, or NLS-R62D actin (non-polymerisable mutant). Distance moved was calculated by measuring the change in scratch area compared to 0 hours and divided by the length of the field of view. Data represent mean ± SEM, *P<0.05 compared to HeLa or nAC controls (N=3-4).

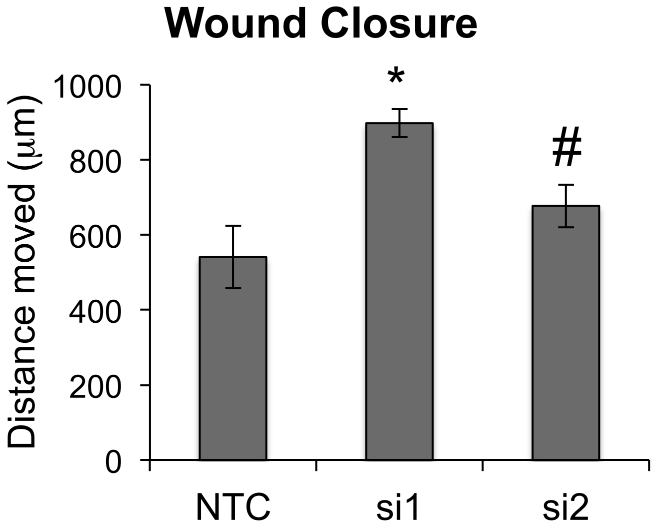

Figure S4: Scratch wound closure with second IPO9 siRNA. HaCaT cells were transfected with two different siRNA sequences for IPO9 (si1 and si2) or non-targeting control RNA (NTC). Confluent monolayers were scratched 48h after transfection and imaged at 0h and 48h after wounding. Distance moved was calculated by measuring the change in scratch area compared to 0 hours and divided by the length of the field of view. Data represent mean ± SEM, *P<0.05 #P=0.057 compared to NTC controls (N=4).

Movie S1: Time-lapse movie of scratch wound closure for parental HaCaT line over 12 h period (10 min/frame).

Movie S2: Time-lapse movie of scratch wound closure for NLS-actin line over 12 h period (10 min/frame).

Movie S3: Time-lapse movie of random cell migration for parental HaCaT line over 18 h period (10 min/frame).

Movie S4: Time-lapse movie of random cell migration for NLS-actin line over 12 h period (10 min/frame).

Table S1: qRT-PCR primer sequences

Gene	Forward	Reverse
MYL9	GAGGATGTGATTCGCAACGC	CCTCATCTGTGAAGCGGTCA
ITGB1	GTAACCAACCGTAGCAAAGGA	TCCCCTGATCTTAATCGCAAAAC
VCL	GAGGCTGAGGTCCGTAAAAT	CTGGCCCAAGATTCTTTGTGT
PAK1	ACCACCAGTGATTGCTCCAC	GCATCTGGTGGAGTGGTGTT
NFATC1	CCATGAAGTCAGCGGAGGAA	GAGGTCTGAAGGTTGTGGCA
BCL2	CTGCACCTGACGCCCTTCACC	CACATGACCCCACCGAACTCAAAGA
GAPDH	AACGGGAAGCTTGTCATCAA	CCCAGCCTTCTCCATGGTG
